# An interpretable Hallmark pathway activity classifier for distinguishing CIN3/HSIL from invasive cervical squamous carcinoma: Development, external validation, and feature stability assessment

**DOI:** 10.1371/journal.pone.0356465

**Published:** 2026-08-28

**Authors:** Mingyu Jia, Youyi Song, Jing Shang, Lijuan Zhuang, Shuhui Xie, Na Cao, Shaofen Ye, Yulian Zhuo, Mingzhu Ye

**Affiliations:** Department of Obstetrics and Gynecology, Zhongshan Hospital Affiliated to Xiamen University, Xiamen, Fujian, China; Mater Olbia Hospital, ITALY

## Abstract

**Background:**

Public transcriptomic cohorts rarely include paired biopsy, conization, and final surgical pathology labels, making direct modeling of post-conization pathological upgrading infeasible in most public datasets. We therefore examined whether pathway-level transcriptomic activity can distinguish CIN3/HSIL from invasive cervical squamous carcinoma.

**Methods:**

GSE63514 was used for model development and GSE7803 as the primary external validation cohort. Expression matrices were mapped to gene symbols and summarized as MSigDB Hallmark pathway activity scores using ssGSEA. An elastic net logistic regression classifier was trained in GSE63514 and applied to GSE7803 without refitting or threshold re-optimization. Bootstrap resampling was used to assess feature-selection stability.

**Results:**

The final model retained eight Hallmark pathways. In GSE63514, the classifier achieved an AUC of 0.890 (95% CI, 0.810–0.971). In GSE7803, the locked model achieved an AUC of 0.815 (95% CI, 0.665–0.966). Bootstrap analysis showed recurrent selection of the major contributing pathways, including estrogen response early, KRAS signaling DN, TGF-beta signaling, estrogen response late, and epithelial-mesenchymal transition.

**Conclusions:**

This study provides an interpretable pathway-level transcriptomic classifier that separates preinvasive high-grade cervical disease from invasive squamous carcinoma in public cohorts. The model should be interpreted as a molecular characterization framework rather than a clinically deployable diagnostic assay.

## Introduction

Cervical cancer remains a major global health burden despite the availability of human papillomavirus (HPV) vaccination and screening programs. Recent global estimates indicate that cervical cancer remains among the most common cancers in women worldwide, with a disproportionate burden in settings where organized screening and timely treatment are limited [1,2]. Persistent infection with carcinogenic HPV types is the necessary causal event in most cervical cancers, but only a subset of infected women progress to high-grade precancer or invasive disease [3].

High-grade squamous intraepithelial lesions, particularly CIN3/HSIL, are immediate precursor lesions of invasive cervical squamous carcinoma and therefore represent a key point for clinical intervention [4,5]. In routine practice, the distinction between high-grade intraepithelial disease and invasive carcinoma is made histopathologically. Histology alone, however, does not fully describe the molecular programs that accompany the transition from preinvasive epithelial disease to invasion. A clearer understanding of this boundary may help characterize invasion-associated states in cervical carcinogenesis.

With the broader implementation of HPV-based screening, the clinical challenge has shifted from detecting HPV infection itself to identifying which HPV-associated abnormalities carry molecular features closer to clinically consequential high-grade or invasive disease [5–7]. Molecular triage strategies, including DNA methylation-based approaches, have therefore attracted increasing attention because they may capture biological changes linked to transforming infection and cancer risk [7]. Although the present study is not a screening or methylation study, this broader shift underscores the need for molecular characterization of cervical precancer and cancer beyond morphology alone.

Transcriptomic profiling offers an opportunity to interrogate these disease states at the molecular level. Many public-data studies have focused on individual differentially expressed genes or large gene-level signatures. Such signatures may show apparent discrimination in a discovery cohort yet remain unstable across platforms and difficult to interpret biologically. A pathway-level representation may reduce dimensionality, summarize coordinated biological programs, and provide a more interpretable model structure [8–11].

The MSigDB Hallmark collection summarizes major biological processes into curated gene sets with reduced redundancy [9]. By transforming expression matrices into sample-level pathway activity scores, disease states can be modeled at the level of biological programs rather than isolated transcripts. In this study, we developed an elastic net classifier based on Hallmark pathway activity scores to distinguish CIN3/HSIL from invasive cervical squamous carcinoma. The model was trained in GSE63514, externally validated in GSE7803, and further examined by bootstrap feature-stability analysis. The goal was not to create a ready-to-use clinical test, but to assess whether a small set of pathway-level features could capture invasion-associated transcriptomic states in public cervical disease cohorts.

## Materials and Methods

### Study design

**Fig 1:**
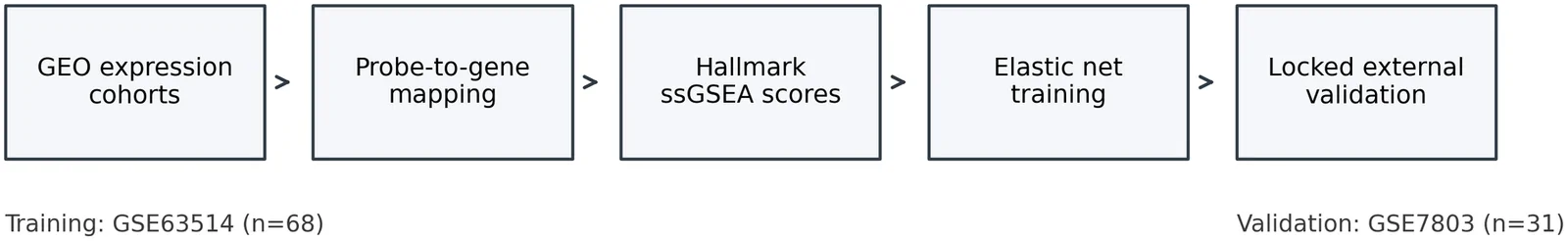
Study workflow. Public GEO expression datasets were processed into gene-level matrices, summarized as Hallmark pathway-activity scores by ssGSEA, and used to train an elastic net logistic regression model. The locked model was then externally validated in GSE7803.

This was a retrospective public-data modeling study. GSE63514 was used for model development, and GSE7803 served as the primary external validation cohort. The trained model was applied to GSE7803 without model refitting, feature reselection, or threshold re-optimization. The overall workflow is summarized in Fig 1. The analysis was reported with reference to general principles for prediction-model reporting and validation [12,13].

### Datasets and analytic cohorts

In GSE63514, samples annotated as CIN3 and invasive cervical cancer were retained. The final training cohort contained 68 samples: 40 CIN3 samples and 28 invasive cancer samples. In GSE7803, samples annotated as HSIL/CIN3 or invasive cervical squamous carcinoma were retained. The final validation cohort contained 31 samples: 7 HSIL/CIN3 samples and 24 invasive squamous carcinoma samples. The unequal class sizes reflected all eligible samples available in the original GEO series and were not created by case-control matching, down-sampling, synthetic augmentation, or class balancing. No a priori sample-size calculation was performed because the cohort sizes were fixed by public data availability. GSE7803 was retained as an independent external cohort despite the limited number of HSIL/CIN3 samples because it provided compatible disease labels on a different microarray platform. Accordingly, validation estimates were reported with 95% confidence intervals and interpreted cautiously. A small RNA-seq dataset reviewed during project development was not used as confirmatory validation because of its limited sample size and platform differences.

### Expression preprocessing

Each dataset was processed separately from GEO-derived expression matrices. For microarray data, probe identifiers were mapped to gene symbols using platform-specific Bioconductor annotation resources. GPL570 probes in GSE63514 were annotated using hgu133plus2.db, and GPL96 probes in GSE7803 were annotated using hgu133a.db. When multiple probes mapped to the same gene symbol, the probe with the greatest variance across samples was retained. Training and validation cohorts were never combined during preprocessing, feature selection, model fitting, or threshold selection.

### Pathway activity scoring

Gene-level expression matrices were transformed into sample-level pathway-activity matrices using single-sample gene set enrichment analysis implemented through the GSVA framework [11]. The MSigDB Hallmark gene set collection was used as the pathway reference [9,10]. The same gene-set file and scoring strategy were applied across cohorts.

### Model development

An elastic net logistic regression model was trained in GSE63514 using Hallmark pathway-activity scores as predictors. Invasive cancer was coded as 1 and CIN3/HSIL as 0. The elastic net mixing parameter was set to alpha = 0.5. Five-fold cross-validation was used, and the final model was selected at lambda.1se to favor a more parsimonious classifier [14,15]. The fitted cv.glmnet object selected at lambda.1se was saved and retained as the locked model for external validation. A fixed threshold of 0.4389156 was taken from the training-cohort model-development output and prespecified before external validation.

### External validation and performance assessment

For GSE7803, Hallmark pathway scores were calculated using the same procedure. The GSE63514-trained model was then applied directly to the validation pathway scores to generate predicted probabilities. Discrimination was assessed by the area under the receiver operating characteristic curve (AUC). AUC 95% confidence intervals were calculated using the DeLong method through pROC [16,17]. Sensitivity, specificity, accuracy, positive predictive value (PPV), and negative predictive value (NPV) were calculated at the fixed threshold. Exact binomial 95% confidence intervals were used for these threshold-based metrics. F1 score was reported as a point estimate.

### Bootstrap feature stability analysis

To evaluate the stability of pathway selection, a bootstrap resampling analysis was conducted in the GSE63514 training cohort. In each iteration, 80% of samples were resampled with replacement, and an elastic net logistic regression model was refitted using the same settings as the primary model, including alpha = 0.5 and lambda.1se selection. This process was repeated 1,000 times. Selection frequency for each Hallmark pathway was calculated as the proportion of valid bootstrap models in which the pathway retained a non-zero coefficient.

### Reproducibility materials

The public source datasets are available from GEO under accession numbers GSE63514 and GSE7803. The author-generated analysis scripts are provided in S2 File and cover public-data preprocessing, probe annotation and Hallmark pathway scoring, use of the locked elastic net model, external validation, bootstrap feature-stability analysis, and generation of analysis outputs. To facilitate direct numerical verification, S3 File provides the processed training-cohort Hallmark scores, analytic sample metadata, the saved locked model object, sample-level external-validation predictions, model coefficients, performance summaries, and bootstrap selection-frequency outputs. The Hallmark gene-set file is obtained separately from MSigDB under its applicable terms and is not redistributed. Detailed analysis parameters are provided in Table A in S1 File; complete performance metrics are provided in Table B in S1 File; and bootstrap stability results are provided in Tables C and D in S1 File.

### Ethics statement

This study used publicly available de-identified transcriptomic datasets from the Gene Expression Omnibus. No new human participants were recruited, no human tissue samples were collected, and no private patient-level clinical records were accessed. Therefore, additional institutional ethics approval and informed consent were not required for this secondary analysis of public data.

## Results

### Analytic cohorts

The training cohort contained 68 samples from GSE63514, including 40 CIN3 samples and 28 invasive cervical cancer samples. The primary external validation cohort contained 31 samples from GSE7803, including 7 HSIL/CIN3 samples and 24 invasive squamous carcinoma samples (Table 1). The analysis workflow from public expression matrices to locked external validation is shown in Fig 1.

**Table 1:** Analytic cohorts and roles in the study.

Cohort	Role	Platform	Analytic task	N	Group composition
GSE63514	Training cohort	Microarray (GPL570)	CIN3 vs invasive cervical cancer	68	40 CIN3; 28 invasive cancer
GSE7803	Primary external validation cohort	Microarray (GPL96)	HSIL/CIN3 vs invasive squamous carcinoma	31	7 HSIL/CIN3; 24 invasive SCC

### Final eight-pathway classifier

**Fig 2:**
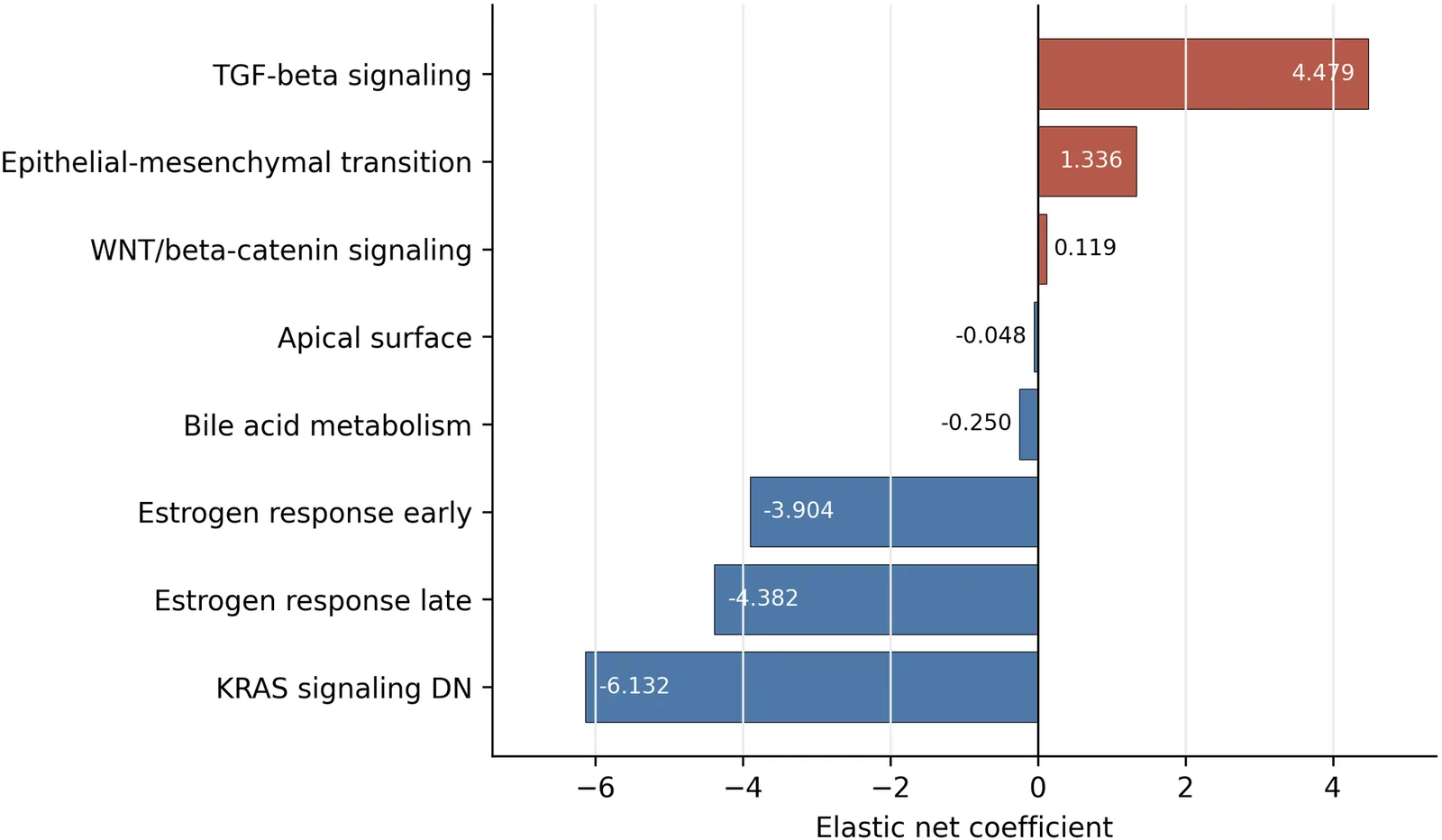
Non-zero coefficients of the final elastic net model. Positive coefficients indicate a higher probability of invasive carcinoma, whereas negative coefficients indicate a lower probability of invasive carcinoma.

**Fig 3:**
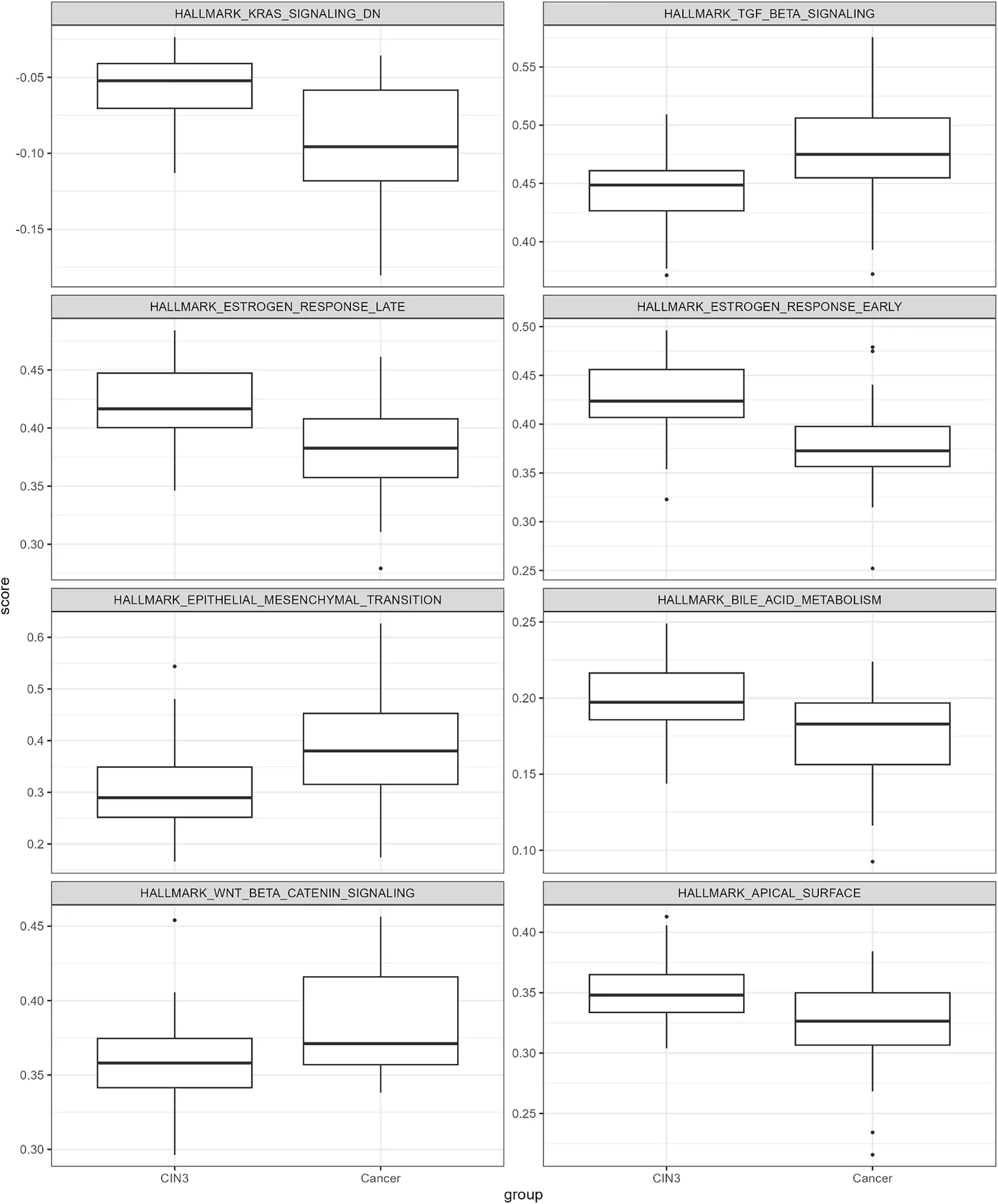
Distribution of selected Hallmark pathway-activity scores between CIN3/HSIL and invasive cervical cancer samples in the training cohort.

At lambda.1se, the elastic net model retained eight non-zero Hallmark pathways (Table 2 and Fig 2). Positive coefficients were assigned to TGF-beta signaling, epithelial-mesenchymal transition, and WNT/beta-catenin signaling, consistent with invasion-associated biological programs. Negative coefficients were observed for KRAS signaling DN, estrogen response early, estrogen response late, bile acid metabolism, and apical surface. The distributions of selected pathway-activity scores in the training cohort are shown in Fig 3.

**Table 2:** Non-zero coefficients of the final elastic net model at lambda.1se.

Hallmark pathway	Coefficient
KRAS signaling DN	−6.132
TGF-beta signaling	4.479
Estrogen response late	−4.382
Estrogen response early	−3.904
Epithelial-mesenchymal transition	1.336
Bile acid metabolism	−0.250
WNT/beta-catenin signaling	0.119
Apical surface	−0.048

### Training cohort performance

**Fig 4:**
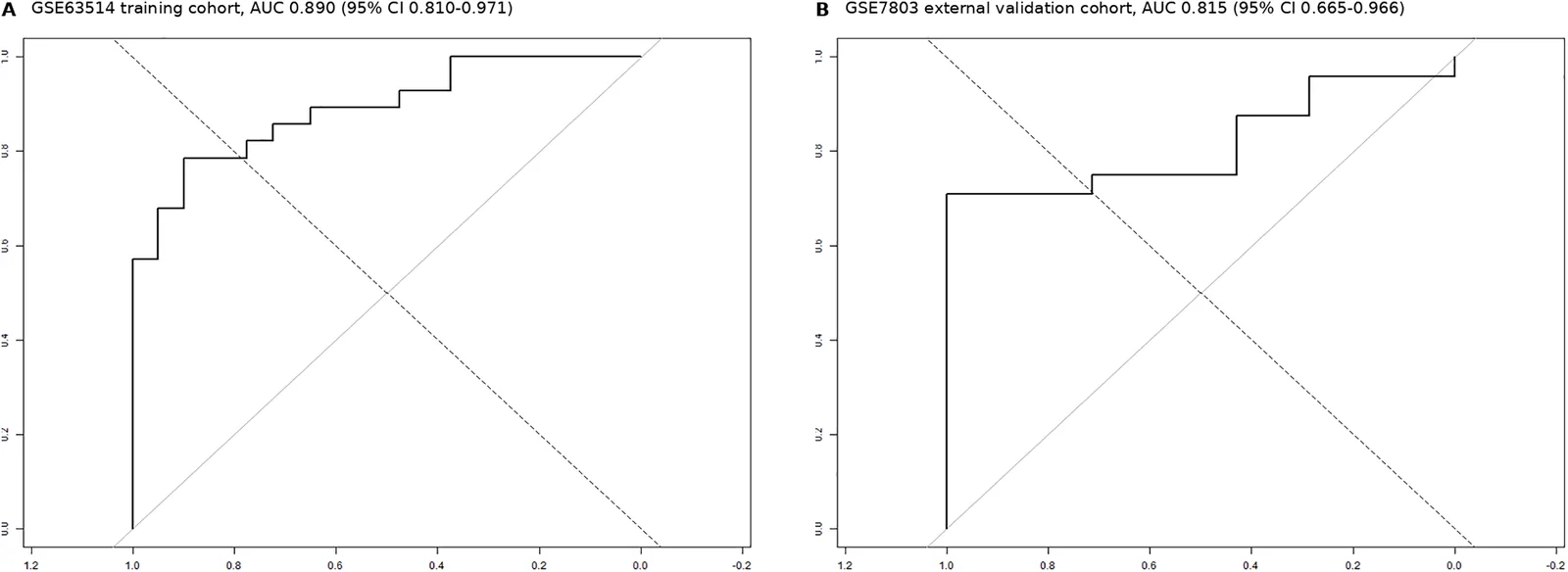
Discrimination of the Hallmark pathway classifier. (A) ROC curve for the GSE63514 training cohort. (B) ROC curve for the GSE7803 primary external validation cohort.

In GSE63514, the pathway-based model distinguished CIN3 from invasive cervical cancer with an AUC of 0.890 (95% CI, 0.810–0.971). At the fixed threshold of 0.4389156, sensitivity was 0.786 (95% CI, 0.590–0.917), specificity was 0.900 (95% CI, 0.763–0.972), and accuracy was 0.853 (95% CI, 0.746–0.927) (Table 3). The training-cohort ROC curve is shown in Fig 4.

**Table 3:** Model performance in the training cohort and primary external validation cohort. AUC confidence intervals were calculated using the DeLong method. Confidence intervals for threshold-based proportions were calculated using exact binomial methods. F1 score is reported as a point estimate.

Cohort	N	AUC (95% CI)	Threshold	Sensitivity (95% CI)	Specificity (95% CI)	Accuracy (95% CI)	PPV (95% CI)	NPV (95% CI)	F1
GSE63514 training	68	0.890 (0.810-0.971)	0.4389	0.786 (0.590-0.917)	0.900 (0.763-0.972)	0.853 (0.746-0.927)	0.846 (0.651-0.956)	0.857 (0.715-0.946)	0.815
GSE7803 validation	31	0.815 (0.665-0.966)	0.4389	0.667 (0.447-0.844)	1.000 (0.590-1.000)	0.742 (0.554-0.881)	1.000 (0.794-1.000)	0.467 (0.213-0.734)	0.800

### Primary external validation in GSE7803

The locked model was then applied to GSE7803 without retraining. In this primary external validation cohort, the AUC was 0.815 (95% CI, 0.665–0.966). Using the same training-derived threshold of 0.4389156, sensitivity was 0.667 (95% CI, 0.447–0.844), specificity was 1.000 (95% CI, 0.590–1.000), and accuracy was 0.742 (95% CI, 0.554–0.881) (Table 3). The external-validation ROC curve is shown in Fig 4. The high specificity and other threshold-based estimates should be interpreted cautiously because the validation cohort was small and included only seven HSIL/CIN3 samples.

### Bootstrap pathway selection stability

Bootstrap pathway-selection analysis was performed to examine feature-selection stability in the GSE63514 training cohort. Among the eight pathways retained in the final classifier, five showed high selection frequencies across 1,000 bootstrap iterations: estrogen response early (88.2%), KRAS signaling DN (84.5%), TGF-beta signaling (81.1%), estrogen response late (80.5%), and epithelial-mesenchymal transition (72.5%). Apical surface showed moderate stability (58.0%), whereas WNT/beta-catenin signaling (35.6%) and bile acid metabolism (32.1%) showed lower selection frequencies. These results indicate that the core structure of the classifier was driven by recurrently selected pathway-level features, whereas low-weight auxiliary pathways should be interpreted more cautiously (Tables C and D in S1 File).

## Discussion

This study developed an interpretable pathway-level transcriptomic classifier for distinguishing CIN3/HSIL from invasive cervical squamous carcinoma. The analysis intentionally avoided the claim that public data can directly predict post-conization pathological upgrading [18]. Instead, it addressed a question that public transcriptomic cohorts can support: whether high-grade preinvasive cervical disease and invasive squamous carcinoma show separable pathway-level molecular states.

The selected pathways provide a biologically plausible explanation for the classifier. TGF-beta signaling, epithelial-mesenchymal transition, and WNT/beta-catenin signaling had positive coefficients, consistent with epithelial plasticity, stromal interaction, and invasion-associated programs. Estrogen-response pathways and KRAS signaling DN had negative coefficients [19], suggesting that the classifier also captured differentiation- or tissue-context-related programs that were relatively stronger in preinvasive lesions.

Bootstrap stability analysis further showed that the principal contributors to the classifier, particularly estrogen response, KRAS signaling DN, TGF-beta signaling, and EMT, were recurrently selected. By contrast, pathways with smaller coefficients showed lower selection frequencies, suggesting that these minor features should be regarded as auxiliary components rather than core biological drivers. This distinction helps prevent overinterpretation of low-weight features while supporting the robustness of the main biological signal.

External validation was the central safeguard against overly optimistic interpretation. The model trained in GSE63514 was not refitted in GSE7803, and the training-derived threshold was applied without optimization in the validation set. The validation AUC remained acceptable, but its confidence interval was wide and threshold-based estimates were imprecise because of the small validation cohort, particularly the seven HSIL/CIN3 samples. The unequal group sizes reflect the available public dataset rather than deliberate case selection. The validation findings therefore provide preliminary cross-platform support but not a precise estimate of clinical performance, and the model should be viewed as a pathway-level molecular characterization framework rather than a diagnostic or triage assay.

Newly collected patient tissue samples were not incorporated because the study was designed as a secondary analysis of public transcriptomic cohorts. Collection and transcriptomic profiling of additional tissue would require a separate clinical protocol, institutional ethics approval, standardized specimen processing, and independent clinicopathological annotation. We agree that such validation would strengthen the evidence base. Future studies should therefore evaluate the locked pathway model in larger, multi-center tissue cohorts and, ideally, in prospectively collected specimens before clinical translation is considered.

From a clinical perspective, transcriptomic analysis may provide molecular context that complements, rather than replaces, cytology and histopathology. Cytology-based screening has substantially reduced the cervical cancer burden, but false-negative or discordant findings can still occur because of sampling limitations, lesion heterogeneity, and interpretive variability. A published case of HPV-53-related cervical cancer following previous negative Pap cytology illustrates that cytology alone may not always capture clinically relevant disease [20]. Although the present analysis used tissue transcriptomic data rather than cytology specimens, pathway-level molecular signals may help characterize the biological boundary between CIN3/HSIL and invasive squamous carcinoma and inform future studies of difficult or discordant diagnostic scenarios.

Importantly, the current classifier should not be interpreted as a replacement for conventional diagnostic assessment. Histopathology remains the reference standard for distinguishing high-grade intraepithelial lesions from invasive carcinoma. The potential value of pathway-level transcriptomic modeling lies in summarizing invasion-associated molecular programs and generating hypotheses for future diagnostic adjuncts. Prospective studies using clinically relevant specimens, including biopsy material or appropriately processed cytology-derived samples, will be required to determine whether such molecular information can reduce misclassification or improve diagnostic confidence.

The study has several limitations. First, the analysis used public tissue-transcriptomic datasets and therefore cannot directly answer whether an individual patient with CIN2/3 will be upgraded after conization. Second, GSE7803 was small and imbalanced, particularly in the HSIL/CIN3 subgroup; the wide confidence intervals and threshold-based estimates should therefore be interpreted cautiously. Third, both main cohorts were microarray datasets, and performance in RNA-seq cohorts or clinically deployable sample types remains uncertain. Fourth, no wet-lab validation or prospective clinical validation was performed. Larger cohorts with linked biopsy, conization, and final pathology data will be required before diagnostic application can be considered.

## Conclusions

An eight-pathway Hallmark-activity classifier distinguished CIN3/HSIL from invasive cervical squamous carcinoma in public transcriptomic cohorts. The model performed well in the training cohort and retained acceptable discrimination in an independent external validation cohort. Bootstrap analysis supported recurrent selection of the major contributing pathways. These findings support the utility of pathway-level transcriptomic modeling for characterizing invasion-associated molecular states in cervical disease, but further validation in larger clinically annotated cohorts is required before diagnostic application can be considered.

### Code availability statement

The path-normalized R scripts documenting and reproducing GEO data preprocessing, probe annotation, Hallmark pathway scoring, elastic net model development, locked external validation, bootstrap feature-stability analysis, and figure and table generation are provided without access restriction as S2 File. The source expression datasets are available from GEO under accession numbers GSE63514 and GSE7803 [21,22].

## Supporting information

S1 File Supplementary tables. This Word file contains Table A, detailed analysis parameters; Table B, complete model performance metrics; Table C, bootstrap selection stability of the eight pathways retained in the final classifier; and Table D, the 15 Hallmark pathways with the highest bootstrap selection frequencies.
(DOCX)

S2 File R analysis scripts. This compressed file contains path-normalized R scripts for reproducing the reported metrics from the shared processed files, implementing the documented elastic net model-fitting procedure, regenerating the locked GSE7803 external validation from public source data, rerunning bootstrap feature-stability analysis, generating summary outputs, and recording session information.
(ZIP)

S3 File Minimal analytic data. This compressed file contains the processed GSE63514 Hallmark pathway-score matrix, analytic sample metadata, the saved locked elastic net model object, sample-level GSE7803 predictions, final model coefficients, training and validation performance outputs, bootstrap selection-frequency results, a README file, and the recorded R session information.
(ZIP)
