# Supplementary material for: An interpretable Hallmark pathway activity classifier for distinguishing CIN3/HSIL from invasive cervical squamous carcinoma: Development, external validation, and feature stability assessment
Source: PLoS One. 2026 Aug 28;21(8):e0356465. doi: 10.1371/journal.pone.0356465 (PMC13524260; doi:10.1371/journal.pone.0356465)
Supplement: Supplementary file 1 [file pone.0356465.s001.docx]

**Supplementary Materials**

An interpretable Hallmark pathway activity classifier for distinguishing CIN3/HSIL from invasive cervical squamous carcinoma: development, external validation, and feature stability assessment

Mingyu Jia, Youyi Song, Jing Shang, Lijuan Zhuang, Shuhui Xie, Na Cao, Shaofen Ye, Yulian Zhuo, Mingzhu Ye

This file contains four supplementary tables documenting analysis parameters, complete model performance metrics, and bootstrap pathway-selection stability results.

**Table A. Detailed analysis parameters.**

| **Parameter** | **Specification** |
| --- | --- |
| Study design | Retrospective public-data transcriptomic modeling study |
| Training cohort | GSE63514; microarray platform GPL570; 68 samples retained (40 CIN3 and 28 invasive cervical cancer) |
| Primary external validation cohort | GSE7803; microarray platform GPL96; 31 samples retained (7 HSIL/CIN3 and 24 invasive cervical squamous carcinoma) |
| Probe-to-gene annotation | GSE63514: hgu133plus2.db; GSE7803: hgu133a.db |
| Multiple-probe handling | When multiple probes mapped to the same gene symbol, the probe with the greatest variance across samples was retained |
| Pathway reference | MSigDB Hallmark gene set collection |
| Pathway scoring method | Single-sample gene set enrichment analysis (ssGSEA) implemented through the GSVA framework |
| Predictors | Sample-level Hallmark pathway activity scores |
| Outcome coding | Invasive carcinoma = 1; CIN3/HSIL = 0 |
| Model | Elastic net logistic regression |
| Elastic net alpha | 0.5 |
| Internal model selection | Five-fold cross-validation |
| Final lambda | lambda.1se |
| Fixed decision threshold | 0.4389156; derived from the training-cohort model-development output and fixed before external validation |
| External validation rule | Locked GSE63514-trained model applied to GSE7803 without refitting, feature reselection, or threshold re-optimization |
| AUC confidence interval method | DeLong method using pROC |
| Threshold-based metric confidence interval method | Exact binomial 95% confidence intervals |
| F1 score | Reported as a point estimate |
| Bootstrap stability analysis | 1,000 bootstrap iterations; in each iteration, 80% of samples were resampled with replacement and the elastic net model was refitted using alpha = 0.5 and lambda.1se |
| Software environment | R package versions and session information were recorded in the reproducibility materials |

**Table B. Complete model performance metrics.**

AUC confidence intervals were calculated using the DeLong method. Confidence intervals for threshold-based proportions were calculated using exact binomial methods. F1 score is reported as a point estimate. Positive class: invasive carcinoma.

| **Cohort** | **N** | **Positive / negative** | **AUC (95% CI)** | **Threshold** | **Sensitivity (95% CI)** | **Specificity (95% CI)** | **Accuracy (95% CI)** | **PPV (95% CI)** | **NPV (95% CI)** | **F1** |
| --- | --- | --- | --- | --- | --- | --- | --- | --- | --- | --- |
| GSE63514 training | 68 | 28 / 40 | 0.890 (0.810-0.971) | 0.4389 | 0.786 (0.590-0.917) | 0.900 (0.763-0.972) | 0.853 (0.746-0.927) | 0.846 (0.651-0.956) | 0.857 (0.715-0.946) | 0.815 |
| GSE7803 validation | 31 | 24 / 7 | 0.815 (0.665-0.966) | 0.4389 | 0.667 (0.447-0.844) | 1.000 (0.590-1.000) | 0.742 (0.554-0.881) | 1.000 (0.794-1.000) | 0.467 (0.213-0.734) | 0.800 |

**Table C. Bootstrap selection stability of the eight Hallmark pathways retained in the final classifier.**

Selection frequency was calculated as the proportion of 1,000 bootstrap elastic net models in which each pathway retained a non-zero coefficient.

| **Hallmark pathway** | **Coefficient** | **Selection frequency** | **Selection frequency (%)** | **Interpretation** |
| --- | --- | --- | --- | --- |
| KRAS signaling DN | -6.132 | 0.845 | 84.5 | High-stability core feature |
| TGF-beta signaling | 4.479 | 0.811 | 81.1 | High-stability core feature |
| Estrogen response late | -4.382 | 0.805 | 80.5 | High-stability core feature |
| Estrogen response early | -3.904 | 0.882 | 88.2 | High-stability core feature |
| Epithelial-mesenchymal transition | 1.336 | 0.725 | 72.5 | High-stability core feature |
| Bile acid metabolism | -0.250 | 0.321 | 32.1 | Lower-stability auxiliary feature |
| WNT/beta-catenin signaling | 0.119 | 0.356 | 35.6 | Lower-stability auxiliary feature |
| Apical surface | -0.048 | 0.580 | 58.0 | Moderate-stability auxiliary feature |

**Table D. Top 15 Hallmark pathways ranked by bootstrap selection frequency.**

| **Rank** | **Hallmark pathway** | **Selection frequency** | **Selection frequency (%)** |
| --- | --- | --- | --- |
| 1 | Estrogen response early | 0.882 | 88.2 |
| 2 | KRAS signaling DN | 0.845 | 84.5 |
| 3 | TGF-beta signaling | 0.811 | 81.1 |
| 4 | Estrogen response late | 0.805 | 80.5 |
| 5 | Epithelial-mesenchymal transition | 0.725 | 72.5 |
| 6 | Apical surface | 0.580 | 58.0 |
| 7 | Angiogenesis | 0.418 | 41.8 |
| 8 | Oxidative phosphorylation | 0.358 | 35.8 |
| 9 | WNT/beta-catenin signaling | 0.356 | 35.6 |
| 10 | Hedgehog signaling | 0.345 | 34.5 |
| 11 | UV response DN | 0.327 | 32.7 |
| 12 | Bile acid metabolism | 0.321 | 32.1 |
| 13 | Interferon alpha response | 0.315 | 31.5 |
| 14 | Androgen response | 0.309 | 30.9 |
| 15 | Protein secretion | 0.260 | 26.0 |
