# Supplementary material for: An interpretable Hallmark pathway activity classifier for distinguishing CIN3/HSIL from invasive cervical squamous carcinoma: Development, external validation, and feature stability assessment
Source: PLoS One. 2026 Aug 28;21(8):e0356465. doi: 10.1371/journal.pone.0356465 (PMC13524260; doi:10.1371/journal.pone.0356465)
Supplement: Supplementary file 4 [file pone.0356465.s004.docx]

**Response to Reviewers**

**Manuscript ID: PONE-D-26-30607**

**Title: An interpretable Hallmark pathway activity classifier for distinguishing CIN3/HSIL from invasive cervical squamous carcinoma: development, external validation, and feature stability assessment**

Dear Dr. Ianieri and Reviewers,

We sincerely thank the Academic Editor and both reviewers for their careful evaluation and constructive comments. We are grateful that both reviewers considered the study technically sound and that Reviewer #2 regarded the pathway-level approach as original and compelling. We have revised the manuscript substantially and provide point-by-point responses below. All changes are highlighted in the marked manuscript, and a clean revised manuscript is also provided.

**Response to Journal Requirements**

**1. Please ensure that your manuscript meets PLOS ONE's style requirements, including those for file naming.**

Response: We reviewed the PLOS ONE formatting and file-naming requirements. The clean manuscript, marked manuscript, response letter, supplementary tables, and R scripts have concise file names and have been assigned the appropriate submission item types.

**2. Please ensure that author-generated code is shared in accordance with PLOS policy.**

Response: We revised the Reproducibility Materials and Code Availability sections. Path-normalized R scripts documenting and reproducing the analysis workflow are now provided without access restriction as S2 File, together with the saved locked model and processed verification data in S3 File. They cover GEO data preprocessing, probe annotation, Hallmark pathway scoring, elastic net model development, locked external validation, bootstrap feature-stability analysis, and generation of figures and tables.

**3. Please ensure that the corresponding author has a validated ORCID iD in Editorial Manager.**

Response: The corresponding author has linked and authenticated the ORCID iD in Editorial Manager.

**4. Please amend the manuscript submission data to include author Shaofen Ye, YuLian and ZhuoMingzhu Ye.**

Response: We corrected the online authorship information so that Shaofen Ye is included and Yulian Zhuo and Mingzhu Ye appear as two separate author records. The online author list now matches the revised manuscript.

**5. Please amend the authorship list in the manuscript file to include MingZhu Ye and YuLian Zhuo.**

Response: The manuscript author list has been corrected to include Yulian Zhuo and Mingzhu Ye as separate authors. The affiliations and Author Contributions statement have also been updated for consistency.

**6. The ethics statement should appear only in the Methods section.**

Response: We moved the Ethics statement to the Materials and Methods section and removed the separate end-matter Ethics statement.

**7. Please refer to Figures 1-4 in the manuscript text.**

Response: We added explicit in-text citations to Fig 1, Fig 2, Fig 3, and Fig 4 in the Materials and Methods and Results sections.

**8. Please include captions for Supporting Information files at the end of the manuscript and update in-text citations.**

Response: We added captions for S1 File (supplementary tables), S2 File (R analysis scripts), and S3 File (minimal analytic data) at the end of the manuscript. The supplementary tables are labeled Tables A-D within S1 File, and the in-text citations have been updated accordingly.

**9. Please evaluate reviewer-recommended references for relevance.**

Response: We reviewed the publication recommended by Reviewer #2 and considered it relevant to the discussion of false-negative or discordant cytology findings. We cited it cautiously as an illustrative clinical report rather than as direct evidence for the performance of our classifier.

**10. Please review the reference list for completeness, correctness, and retracted articles.**

Response: We reviewed and updated the reference list, added the reviewer-recommended article, corrected the subsequent numbering, and found no retracted articles in the revised reference list.

**Response to Additional Editor Comments**

**Comment: The small sample size of GSE7803 should be better explained in the Materials and Methods section, and the Discussion should incorporate the reviewers' suggestions.**

Response: We clarified in Materials and Methods that all eligible samples in the public datasets were retained; the unequal class sizes were not produced by matching, down-sampling, synthetic augmentation, or class balancing; and no a priori sample-size calculation was possible because cohort sizes were fixed by public data availability. We explicitly state that GSE7803 contained only seven HSIL/CIN3 samples and that validation estimates should be interpreted with their confidence intervals. The Discussion has been expanded to address the limited precision of the external validation, the rationale for not collecting new tissue samples within this public-data study, the potential complementary role of transcriptomic information in difficult diagnostic settings, and the need for larger multi-center validation.

**Response to Reviewer #1**

**Comment 1: Why were there not equal numbers in the cohorts? There were only seven CIN3 cases in the validation set, and the sample numbers are very small.**

Response: We thank the reviewer for this important concern. The unequal class sizes reflect all eligible samples available in GSE63514 and GSE7803 and were not chosen by the authors through matching or selective down-sampling. GSE7803 was retained because it provided an independent cohort containing both HSIL/CIN3 and invasive squamous carcinoma on a different microarray platform. We agree that seven HSIL/CIN3 samples are insufficient for precise threshold-based performance estimates. We therefore retained all eligible samples, avoided refitting or threshold optimization in GSE7803, reported AUC and threshold-based metrics with 95% confidence intervals, and strengthened the cautionary interpretation in the Methods, Results, and Discussion. The revised manuscript now explicitly states that the validation is preliminary and does not establish clinical diagnostic performance.

**Comment 2: Why were patient tissue samples not considered? Adding such samples would strengthen the study.**

Response: We agree that independent validation using newly collected patient tissue would strengthen the study. The present work was designed as a secondary analysis of public transcriptomic cohorts. New tissue collection and profiling would require a separate clinical protocol, ethics approval, standardized specimen processing, and independent clinicopathological annotation and was therefore outside the scope of this public-data study. We have added this rationale to the Discussion and now identify larger multi-center tissue cohorts and prospective specimen validation as the principal next steps. We also emphasize that the present classifier is a molecular characterization framework rather than a clinically deployable assay.

**Response to Reviewer #2**

**Comment: The Discussion is too brief. Please discuss the potential for transcriptomic analysis to reduce cytology misdiagnosis, citing Zappacosta et al. (2014; doi:10.2147/CIA.S57294), and its ability to differentiate CIN3/HSIL from squamous cell carcinoma.**

Response: We sincerely thank the reviewer for the positive assessment and helpful suggestion. We substantially expanded the Discussion. The new text explains that transcriptomic pathway information may complement, but cannot replace, cytology and histopathology; discusses sampling limitations, lesion heterogeneity, and interpretive variability as possible sources of false-negative or discordant findings; and cites the suggested HPV-53-related cervical cancer report following negative Pap cytology. We also clarify that the cited report is an illustrative clinical example and that our tissue-based transcriptomic classifier has not been tested directly in cytology specimens. The revised Discussion therefore frames molecular analysis as a future diagnostic adjunct that requires prospective validation.

**Data availability concern:**

Response: Reviewer #2 indicated that the underlying data were not fully available. We clarified that the source expression datasets are publicly available under GSE63514 and GSE7803. S1 File provides detailed parameters and summary numerical outputs; S2 File provides the path-normalized R analysis scripts; and S3 File provides processed sample-level analytic data, the saved locked model object, sample-level external-validation predictions, final coefficients, performance outputs, and bootstrap selection-frequency results. The Data Availability, Reproducibility Materials, and Code Availability statements have been revised accordingly.

We again thank the Academic Editor and reviewers for their time and constructive guidance. We believe the revisions have strengthened the transparency, reproducibility, clinical context, and limitations of the manuscript.

Sincerely,

Mingyu Jia, on behalf of all authors

Mingzhu Ye, Corresponding Author
