# Supplementary material for: An interpretable Hallmark pathway activity classifier for distinguishing CIN3/HSIL from invasive cervical squamous carcinoma: Development, external validation, and feature stability assessment
Source: PLoS One. 2026 Aug 28;21(8):e0356465. doi: 10.1371/journal.pone.0356465 (PMC13524260; doi:10.1371/journal.pone.0356465)
Supplement: Peer review history

# Peer review: Decision Letter 0

**DOI:** 10.1371/journal.pone.0356465.r001

**Authors:** Manuel Maria Ianieri

8 Jul 2026

Dear Dr. Ye,

Thank you for submitting your manuscript to PLOS ONE. After careful consideration, we feel that it has merit but does not fully meet PLOS ONE’s publication criteria as it currently stands. Therefore, we invite you to submit a revised version of the manuscript that addresses the points raised during the review process.

Please submit your revised manuscript by Aug 07 2026 11:59PM. If you will need more time than this to complete your revisions, please reply to this message or contact the journal office at plosone@plos.org. When you're ready to submit your revision, log on to https://www.editorialmanager.com/pone/ and select the 'Submissions Needing Revision' folder to locate your manuscript file.

- A letter that responds to each point raised by the academic editor and reviewer(s). You should upload this letter as a separate file labeled 'Response to Reviewers'.
- A marked-up copy of your manuscript that highlights changes made to the original version. You should upload this as a separate file labeled 'Revised Manuscript with Track Changes'.
- An unmarked version of your revised paper without tracked changes. You should upload this as a separate file labeled 'Manuscript'.

If you would like to make changes to your financial disclosure, please include your updated statement in your cover letter. Guidelines for resubmitting your figure files are available below the reviewer comments at the end of this letter.

If applicable, we recommend that you deposit your laboratory protocols in protocols.io to enhance the reproducibility of your results. Protocols.io assigns your protocol its own identifier (DOI) so that it can be cited independently in the future. For instructions see: https://journals.plos.org/plosone/s/submission-guidelines#loc-laboratory-protocols. Additionally, PLOS ONE offers an option for publishing peer-reviewed Lab Protocol articles, which describe protocols hosted on protocols.io. Read more information on sharing protocols at https://plos.org/protocols?utm_medium=editorial-email&utm_source=authorletters&utm_campaign=protocols.

As the corresponding author, your ORCID iD is verified in the submission system and will appear in the published article. PLOS supports the use of ORCID, and we encourage all coauthors to register for an ORCID iD and use it as well. Please encourage your coauthors to verify their ORCID iD within the submission system before final acceptance, as unverified ORCID iDs will not appear in the published article. *Only*  the individual author can complete the verification step; PLOS staff *cannot*  verify ORCID iDs on behalf of authors.

We look forward to receiving your revised manuscript.

Kind regards,

Manuel Maria Ianieri, M.D.; Ph.D.

Academic Editor

PLOS One

Journal Requirements:

When submitting your revision, we need you to address these additional requirements.

1. Please ensure that your manuscript meets PLOS ONE's style requirements, including those for file naming. The PLOS ONE style templates can be found at

https://journals.plos.org/plosone/s/file?id=wjVg/PLOSOne_formatting_sample_main_body.pdf and

https://journals.plos.org/plosone/s/file?id=ba62/PLOSOne_formatting_sample_title_authors_affiliations.pdf

2. Please note that PLOS One has specific guidelines on code sharing for submissions in which author-generated code underpins the findings in the manuscript. In these cases, we expect all author-generated code to be made available without restrictions upon publication of the work. Please review our guidelines at https://journals.plos.org/plosone/s/materials-and-software-sharing#loc-sharing-code and ensure that your code is shared in a way that follows best practice and facilitates reproducibility and reuse.

3. PLOS requires an ORCID iD for the corresponding author in Editorial Manager on papers submitted after December 6th, 2016. Please ensure that you have an ORCID iD and that it is validated in Editorial Manager. To do this, go to ‘Update my Information’ (in the upper left-hand corner of the main menu), and click on the Fetch/Validate link next to the ORCID field. This will take you to the ORCID site and allow you to create a new iD or authenticate a pre-existing iD in Editorial Manager.

4. Please amend the manuscript submission data (via Edit Submission) to include author Shaofen Ye, YuLian and ZhuoMingzhu Ye.

5. Please amend your authorship list in your manuscript file to include author MingZhu Ye and YuLian Zhuo.

6. Your ethics statement should only appear in the Methods section of your manuscript. If your ethics statement is written in any section besides the Methods, please move it to the Methods section and delete it from any other section. Please ensure that your ethics statement is included in your manuscript, as the ethics statement entered into the online submission form will not be published alongside your manuscript.

7. Please ensure that you refer to Figures 1 - 4 in your text as, if accepted, production will need this reference to link the reader to the figure.

8. Please include captions for your Supporting Information files at the end of your manuscript, and update any in-text citations to match accordingly. Please see our Supporting Information guidelines for more information: http://journals.plos.org/plosone/s/supporting-information.

9. If the reviewer comments include a recommendation to cite specific previously published works, please review and evaluate these publications to determine whether they are relevant and should be cited. There is no requirement to cite these works unless the editor has indicated otherwise.

10. Please review your reference list to ensure that it is complete and correct. If you have cited papers that have been retracted, please include the rationale for doing so in the manuscript text, or remove these references and replace them with relevant current references. Any changes to the reference list should be mentioned in the rebuttal letter that accompanies your revised manuscript. If you need to cite a retracted article, indicate the article’s retracted status in the References list and also include a citation and full reference for the retraction notice.

Additional Editor Comments:

Dear Author,

I would like to commend you on your interesting study; however, issues raised by the reviewers mean that the study is not yet acceptable for publication.

In particular, I agree with the concerns regarding the small sample size of the GSE7803 dataset, which should be better explained in the Materials and Methods section. Furthermore, the Discussion could be improved by incorporating the suggestions and points for reflection provided.We look forward to your corrections.

[Note: HTML markup is below. Please do not edit.]

Reviewers' comments:

Reviewer's Responses to Questions

**Comments to the Author**

1. Is the manuscript technically sound, and do the data support the conclusions?

Reviewer #1: Yes

Reviewer #2: Yes

**********

2. Has the statistical analysis been performed appropriately and rigorously?

Reviewer #1: No

Reviewer #2: Yes

**********

3. Have the authors made all data underlying the findings in their manuscript fully available?

The PLOS Data policy

Reviewer #1: Yes

Reviewer #2: No

**********

4. Is the manuscript presented in an intelligible fashion and written in standard English?

Reviewer #1: Yes

Reviewer #2: Yes

**********

Reviewer #1: Thank you for submitting this manuscript.

The authors state: "In GSE63514, samples annotated as CIN3 and invasive cervical cancer were retained. The final

training cohort contained 68 samples: 40 CIN3 samples and 28 invasive cancer samples. In

GSE7803, samples annotated as HSIL/CIN3 or invasive cervical squamous carcinoma were

retained. The final validation cohort contained 31 samples: 7 HSIL/CIN3 samples and 24

invasive squamous carcinoma samples." Why were there not equal numbers for the training and validation cohorts? The fact that there were ONLY 7 CIN 3 cases really impacts on results especially for a final validation set. The numbers are very small.

The authors have stated: "The validation AUC remained acceptable, but the confidence interval was wide and threshold-based estimates were imprecise because of the small validation cohort. Testing of more cases is suggested. What was the rationale in NOT considering use of patient tissue samples? Surely adding in this component will strengthen the study?

Reviewer #2: The proposed study may appeal to a niche audience, but it is highly interesting. It focuses on the use of transcriptomic analysis for the differential diagnosis between a pre-invasive lesion, such as CIN3, and squamous cell carcinoma.

I find the study well-structured: the materials and methods are clear and well-described, and the results appear to be presented clearly, accompanied by representative tables.

The study's greatest strength lies in the originality of the proposed approach, which I find very compelling.

However, one section of the article—the discussion—needs improvement as it is currently too brief. I suggest introducing additional points for consideration: specifically, I would discuss the potential for transcriptomic analysis to prevent the misdiagnosis of cytology samples—as documented in extensive literature (citing, for example, Zappocosta R et al. 2014; DOI 10.2147/CIA.S57294)—and its ability to differentiate between a diagnosis of CIN3/HSIL and squamous cell carcinoma.

**********

what does this mean?). If published, this will include your full peer review and any attached files.

If you choose “no”, your identity will remain anonymous but your review may still be made public.

**Do you want your identity to be public for this peer review?**  For information about this choice, including consent withdrawal, please see our Privacy Policy

Reviewer #1: No

Reviewer #2: No

**********

[NOTE: If reviewer comments were submitted as an attachment file, they will be attached to this email and accessible via the submission site. Please log into your account, locate the manuscript record, and check for the action link "View Attachments". If this link does not appear, there are no attachment files.]

To ensure your figures meet our technical requirements, please review our figure guidelines: https://journals.plos.org/plosone/s/figures

You may also use PLOS’s free figure tool, NAAS, to help you prepare publication quality figures: https://journals.plos.org/plosone/s/figures#loc-tools-for-figure-preparation.

NAAS will assess whether your figures meet our technical requirements by comparing each figure against our figure specifications.

# Author comment: Author response to Decision Letter 1

**DOI:** 10.1371/journal.pone.0356465.r002

30 Jul 2026

Dear Academic Editor and Reviewers,

Thank you very much for your careful evaluation of our manuscript and for the constructive comments. We have revised the manuscript thoroughly and addressed all editor and reviewer comments point by point in the attached “Response to Reviewers” document.

The main revisions include: (1) clarifying the rationale for the unequal cohort sizes and the limited number of HSIL/CIN3 samples in GSE7803; (2) expanding the Discussion to address the absence of newly collected patient tissue samples, the limitations of the external validation cohort, and the potential role of transcriptomic analysis as a complement to cytology and histopathology; (3) adding the publication suggested by Reviewer #2; (4) moving the ethics statement to the Materials and Methods section; (5) adding in-text citations for Figs 1–4 and captions for all Supporting Information files; (6) updating the author list and contribution statement as requested; and (7) providing the R analysis scripts and minimal analytic data as Supporting Information to improve reproducibility and data availability.

A clean revised manuscript and a version with changes highlighted have also been uploaded.

We sincerely appreciate the editor’s and reviewers’ comments, which have helped us substantially improve the manuscript.

Attachment Submitted filename: PONE-D-26-30607_Response_to_Reviewers.docx

# Editor report: Decision Letter 1

**DOI:** 10.1371/journal.pone.0356465.r003

**Authors:** Manuel Maria Ianieri

4 Aug 2026

An interpretable Hallmark pathway activity classifier for distinguishing CIN3/HSIL from invasive cervical squamous carcinoma: development, external validation, and feature stability assessment

PONE-D-26-30607R1

Dear Dr. MingZhu

We’re pleased to inform you that your manuscript has been judged scientifically suitable for publication and will be formally accepted for publication once it meets all outstanding technical requirements.

Within one week, you’ll receive an e-mail detailing the required amendments. When these have been addressed, you’ll receive a formal acceptance letter and your manuscript will be scheduled for publication.

An invoice will be generated when your article is formally accepted. Please note, if your institution has a publishing partnership with PLOS and your article meets the relevant criteria, all or part of your publication costs will be covered. Please make sure your user information is up-to-date by logging into Editorial Manager at Editorial Manager® and clicking the ‘Update My Information' link at the top of the page. For questions related to billing, please contact billing support.

If your institution or institutions have a press office, please notify them about your upcoming paper to help maximize its impact. If they’ll be preparing press materials, please inform our press team as soon as possible -- no later than 48 hours after receiving the formal acceptance. Your manuscript will remain under strict press embargo until 2 pm Eastern Time on the date of publication. For more information, please contact onepress@plos.org.

Kind regards,

Manuel Maria Ianieri, M.D.; Ph.D.

Academic Editor

PLOS One

Additional Editor Comments (optional):

Dear Author, we appreciate the effort you put into the review, which appears to be in line with the reviewers' requests. The study appears interesting, and I believe it could be included in the current literature.

# Editor report: Acceptance letter

**DOI:** 10.1371/journal.pone.0356465.r004

**Authors:** Manuel Maria Ianieri

PONE-D-26-30607R1

PLOS One

Dear Dr. Ye,

I'm pleased to inform you that your manuscript has been deemed suitable for publication in PLOS One. Congratulations! Your manuscript is now being handed over to our production team.

At this stage, our production department will prepare your paper for publication. This includes ensuring the following:

* All references, tables, and figures are properly cited

* All relevant supporting information is included in the manuscript submission,

* There are no issues that prevent the paper from being properly typeset

You will receive further instructions from the production team, including instructions on how to review your proof when it is ready. Please keep in mind that we are working through a large volume of accepted articles, so please give us a few days to review your paper and let you know the next and final steps.

Lastly, if your institution or institutions have a press office, please let them know about your upcoming paper now to help maximize its impact. If they'll be preparing press materials, please inform our press team within the next 48 hours. Your manuscript will remain under strict press embargo until 2 pm Eastern Time on the date of publication. For more information, please contact onepress@plos.org.

You will receive an invoice from PLOS for your publication fee after your manuscript has reached the completed accept phase. If you receive an email requesting payment before acceptance or for any other service, this may be a phishing scheme. Learn how to identify phishing emails and protect your accounts at https://explore.plos.org/phishing.

If we can help with anything else, please email us at customercare@plos.org.

Thank you for submitting your work to PLOS One and supporting open access.

Kind regards,

PLOS ONE Editorial Office Staff

on behalf of

Dr. Manuel Maria Ianieri

Academic Editor

PLOS One
